# Large-scale transient peri-ictal perfusion magnetic resonance imaging abnormalities detected by quantitative image analysis

**DOI:** 10.1093/braincomms/fcad047

**Published:** 2023-02-24

**Authors:** Manuel Köstner, Michael Rebsamen, Piotr Radojewski, Christian Rummel, Baudouin Jin, Raphael Meier, Uzeyir Ahmadli, Kaspar Schindler, Roland Wiest

**Affiliations:** Support Center for Advanced Neuroimaging (SCAN), University Institute of Diagnostic and Interventional Neuroradiology, University of Bern, Inselspital, Bern University Hospital, Bern CH-3010, Switzerland; Faculty of Medicine, University of Bern, Bern CH-3008, Switzerland; Support Center for Advanced Neuroimaging (SCAN), University Institute of Diagnostic and Interventional Neuroradiology, University of Bern, Inselspital, Bern University Hospital, Bern CH-3010, Switzerland; Graduate School for Cellular and Biomedical Sciences, University of Bern, Bern CH-3012, Switzerland; Support Center for Advanced Neuroimaging (SCAN), University Institute of Diagnostic and Interventional Neuroradiology, University of Bern, Inselspital, Bern University Hospital, Bern CH-3010, Switzerland; Translational Imaging Center (TIC), sitem-Insel, Bern University Hospital, Bern CH-3010, Switzerland; Support Center for Advanced Neuroimaging (SCAN), University Institute of Diagnostic and Interventional Neuroradiology, University of Bern, Inselspital, Bern University Hospital, Bern CH-3010, Switzerland; Department of Neurology, Inselspital, Sleep-Wake-Epilepsy-Center, Bern University Hospital, University of Bern, Bern CH-3010, Switzerland; Support Center for Advanced Neuroimaging (SCAN), University Institute of Diagnostic and Interventional Neuroradiology, University of Bern, Inselspital, Bern University Hospital, Bern CH-3010, Switzerland; Support Center for Advanced Neuroimaging (SCAN), University Institute of Diagnostic and Interventional Neuroradiology, University of Bern, Inselspital, Bern University Hospital, Bern CH-3010, Switzerland; Department of Neurology, Inselspital, Sleep-Wake-Epilepsy-Center, Bern University Hospital, University of Bern, Bern CH-3010, Switzerland; Support Center for Advanced Neuroimaging (SCAN), University Institute of Diagnostic and Interventional Neuroradiology, University of Bern, Inselspital, Bern University Hospital, Bern CH-3010, Switzerland; Translational Imaging Center (TIC), sitem-Insel, Bern University Hospital, Bern CH-3010, Switzerland

**Keywords:** epilepsy, ictal, perfusion MRI, quantitative MRI, transient peri-ictal perfusion abnormalities TPMA

## Abstract

Epileptic seizures require a rapid and safe diagnosis to minimize the time from onset to adequate treatment. Some epileptic seizures can be diagnosed clinically with the respective expertise. For more subtle seizures, imaging is mandatory to rule out treatable structural lesions and potentially life-threatening conditions. MRI perfusion abnormalities associated with epileptic seizures have been reported in CT and MRI studies. However, the interpretation of transient peri-ictal MRI abnormalities is routinely based on qualitative visual analysis and therefore reader dependent. In this retrospective study, we investigated the diagnostic yield of visual analysis of perfusion MRI during ictal and postictal states based on comparative expert ratings in 51 patients. We further propose an automated semi-quantitative method for perfusion analysis to determine perfusion abnormalities observed during ictal and postictal MRI using dynamic susceptibility contrast MRI, which we validated on a subcohort of 27 patients. The semi-quantitative method provides a parcellation of 3D T_1_-weighted images into 32 standardized cortical regions of interests and subcortical grey matter structures based on a recently proposed method, direct cortical thickness estimation using deep learning–based anatomy segmentation and cortex parcellation for brain anatomy segmentation. Standard perfusion maps from a Food and Drug Administration–approved image analysis tool (Olea Sphere 3.0) were co-registered and investigated for region-wise differences between ictal and postictal states. These results were compared against the visual analysis of two readers experienced in functional image analysis in epilepsy. In the ictal group, cortical hyperperfusion was present in 17/18 patients (94% sensitivity), whereas in the postictal cohort, cortical hypoperfusion was present only in 9/33 (27%) patients while 24/33 (73%) showed normal perfusion. The (semi-)quantitative dynamic susceptibility contrast MRI perfusion analysis indicated increased thalamic perfusion in the ictal cohort and hypoperfusion in the postictal cohort. Visual ratings between expert readers performed well on the patient level, but visual rating agreement was low for analysis of subregions of the brain. The asymmetry of the automated image analysis correlated significantly with the visual consensus ratings of both readers. We conclude that expert analysis of dynamic susceptibility contrast MRI effectively discriminates ictal versus postictal perfusion patterns. Automated perfusion evaluation revealed favourable interpretability and correlated well with the classification of the visual ratings. It may therefore be employed for high-throughput, large-scale perfusion analysis in extended cohorts, especially for research questions with limited expert rater capacity.

## Teaser

Köstner *et al*. analysed MRI perfusion data in patients with seizures and proposed an automated method for quantitative assessment of regional perfusion abnormalities. The analysis revealed thalamic hyperperfusion in ictal patients compared to a postictal cohort that showed hypoperfusion in the thalamus.

## Graphical Abstract

**Graphical Abstract:**
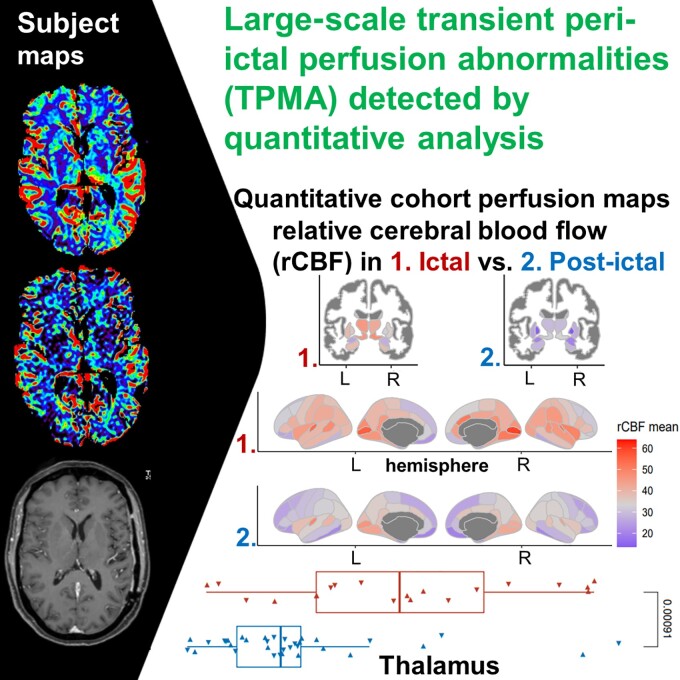

## Introduction

Patients who underwent an epileptic seizure frequently present with unspecific neurologic symptoms mimicking other conditions related to e.g. stroke, migraine, syncope or brain tumours that hamper accurate diagnosis under emergency conditions.^1^ Epileptic seizures are the most frequent presentation in large emergency departments^1^ if they present with (i) ongoing seizure activity and increased metabolic demand^2^ or (ii) focal neurological deficits due to reduced oxygen pressure and cerebral blood flow (CBF).^3^ While detection of ongoing seizure activity or non-convulsive status epilepticus (SE) is mandatory for early antiepileptic treatment,^4,5^ presence of postictal neurological deficits requires further diagnostic workup to rule out underlying factors and complications.^6^

Beyond structural neuroimaging with CT, MRI has become increasingly available for emergency diagnostic workup of transient peri-ictal abnormalities in tertiary care centres and provides access to advanced peri-ictal neuroimaging markers, such as diffusion-weighted imaging (DWI),^7,8^ susceptibility-weighted imaging (SWI)^9,10^ and perfusion imaging.^11–15^

The highest diagnostic yield of advanced neuroimaging for transient peri-ictal MRI abnormalities (TPMA) has been achieved in patients with persistent seizure activity at the time of image acquisition and after repetitive seizures, although peri-ictal abnormalities have also been observed after single events.^11,16,17^ DWI abnormalities are more frequently observed in patients with repetitive seizures or during SE,^18^ and their patterns may overlap with other conditions e.g. encephalitis or during metabolic derangements.^19^ SWI enables the depiction of alterations in venous deoxyhaemoglobin levels, resulting in pseudo-calibre changes of the affected cortical veins during and after an epileptic seizure.^9^ However, SWI is susceptible to extrinsic factors such as mechanical ventilation, haemorrhages and calcification, and hardware-related effects, such as in-plane image distortion and local hypo- and hyperintensity due to non-uniformity of the main magnetic field. In previous studies, perfusion imaging has been demonstrated to be robust and yielded a higher sensitivity than DWI and SWI to depict TPMA.^10^

Since the majority of perfusion studies are performed within clinical stroke protocols, dynamic susceptibility contrast (DSC) MRI is predominantly applied to investigate TPMA.^20^ Several studies have indicated that CBF and cerebral blood volume (CBV) are most sensitive for visual, expert-based analysis of TPMA.^21,22^ While quantitative image analysis has become a standard procedure for the ischaemic core and penumbra in acute ischaemic stroke, quantitative perfusion analysis has not been routinely used for image analysis in epilepsy, mainly due to the lack of critical perfusion thresholds and delayed image acquisitions that hamper routine applications of perfusion imaging. In this study, we propose a novel and standardized approach to analyse TPMA, using subject-wise normalized perfusion metrics as an intra-individual reference. To facilitate automatic quantitative analysis and enable cohort-wide evaluation of perfusion data, we propose a method for extracting region of interest (ROI)-wise statistics defined by anatomical areas derived from a deep learning–based segmentation of structural MR images.^23^ Finally, we contrast these quantitative results to corresponding visual ratings from two neuroradiologists.

We hypothesize that quantitative imaging analysis discriminates ictal and post-ictal TPMA patterns by region-wise perfusion abnormalities in cortical and subcortical regions. We further hypothesize that large-scale perfusion abnormalities can be detected by automated analysis of perfusion imaging using deep learning–based parcellations and subject-wise normalization of TPMA maps, otherwise undetected by visual analysis.

## Materials and methods

### Study type

This retrospective study was approved by the Swiss Ethics Committee (Ref. 2017-00697). Data analysis was performed with anonymized data only. Structural and perfusion MRI were acquired according to the locally implemented clinical protocol to differentiate between ischaemic stroke, epileptic seizures and other stroke mimics.

### Patient cohort

The cohort was defined by conducting a keyword search in the database of the University Institute of Diagnostic and Interventional Neuroradiology, for patients who underwent MR imaging at the University Hospital Bern with the keywords ‘epilepsy’ and ‘perfusion’ in the radiological reports. The search encompassed a period between February 2008 and February 2018. Demographic data, clinical characteristics, imaging reports and final reports were retrieved from the electronic medical records for review.

Patients with neurological deficits due to epileptic seizures, ongoing epileptic activity in EEG (SE or seizure clusters), or patients who underwent MR perfusion imaging within 24 h after a witnessed or drug-interrupted epileptic seizure were enrolled in the analysis. Patients were excluded if the final diagnosis was not an epileptic seizure or the required clinical information was not available in the clinical system.

### Seizure categories and collected variables

Clinical diagnosis followed the International League Against Epilepsy (ILAE) and the International Bureau for Epilepsy (IBE) definition of an epileptic seizure as abnormal excessive or synchronous neuronal activity in the brain causing a transient occurrence of neurological signs and/or symptoms.^24^ SE was defined as a continuous clinical or electrographic seizure activity with a duration of over 5 min or a recurrent seizure activity without recovery between the seizures.^25^ Acute repetitive seizures (seizure clusters) were defined as the presence of three or more seizures occurring within 24 h and regained consciousness between seizures and with no SE in the EEG.^26^

Patients were retrospectively categorized into three cohorts by two authors, using the clinical reports: (i) single seizure, (ii) seizure clusters and (iii) SE encompassing either convulsive SE or non-convulsive SE (NCSE). Seizure onset was categorized according to the ILAE seizure classification provided in the final clinical reports.^27^ Patients presented with either focal, primary or secondary generalized seizures or were classified as of unknown origin.

Patients were classified as ictal at the time of MR imaging (i) during a state of clinically suspected SE or ongoing seizure activity that resolved after administration of antiepileptic treatment according to the guidelines of the ILAE,^28^ which are applied in our hospital, or (ii) via ongoing epileptic activity confirmed by EEG with epileptiform discharges (ED), such as spikes, sharp waves, polyspikes and sharply contoured waves according to the American Clinical Neurophysiology Society’s (ACNS) Standardized Critical Care EEG Terminology.^29^ Patients with terminated witnessed seizures or postictal neurological deficits or absence of epileptic discharges during EEG were classified as postictal. In patients without EEG, the classification was based on the integrative final clinical diagnosis according to the patient’s health records and follow-up. All clinical and radiological data were reviewed by two authors (M.K. and R.W. in consensus). Patients were excluded if clinical reports lacked a final categorization according to the ILAE classification.^27^

To analyse the frequency of reported MRI abnormalities, radiological reports from the subjects were screened for reported restricted diffusion in accordance with the seizure symptoms in DWI sequences and for reported abnormalities in perfusion-weighted images. Reported abnormalities in perfusion-weighted images were classified into either normal perfusion, decreased (hypo-) or increased (hyper-) perfusion.

The interval between the time of admission and the time of MRI acquisition was determined as the intervals between admission according to the logs and the start of the MRI exam. An approximate interval between the first onset of symptoms and MRI acquisition was estimated from medical reports. In patients with unknown seizure onset time, the time last seen well was used for estimation.

### Perfusion image acquisition and evaluation

Images were acquired on a subset of four MRI scanners, as available during 24/7 emergencies from 2008 to 2018: two 1.5 T systems (Magnetom Aera/Avanto) and two 3 T systems (Magnetom Verio and Prisma) from the same vendor (Siemens Healthineers, Erlangen, Germany). The MRI protocols followed our in-house stroke and emergency protocols with the following sequences as a minimum standard: axial DWI, axial T_2_-weighted or fluid-attenuated inversion recovery (FLAIR), time-of-flight angiography (ToF), DSC-perfusion, first-pass contrast-enhanced MR angiography of the head and neck arteries, and axial T_1_-weighted SE post-contrast. Sequence parameters were scanner dependent and varied over time due to adoptions in emergency protocols. A subset of patients who received 3D T_1_-weighted MPRAGE were eligible for (semi-)quantitative image analysis.

DSC-MRI was acquired with a 2D EPI Siemens product sequence for perfusion analysis in all patients (FoV read 230 mm, FoV phase 100%, voxel size 1.8 × 1.8 × 5.0 mm, flip angle 90°, 80 repetitions) following an injection of 0.1 mmol/kg of gadolinium contrast agent with a flow rate of 5 mL/s. Perfusion data were processed with *Olea Sphere 3.0* software (Olea Medical, La Ciotat, France) that has received FDA 510(k) clearance to market, using an automated arterial input function (AIF) selection and singular value decomposition (SVD) deconvolution to generate colour-coded perfusion maps: relative CBF (rCBF), relative CBV (rCBV), mean transit time (MTT), time to peak (TTP), time maximum intensity projection (tMIP) and time to maximum (Tmax).

### Quantitative analysis of patients with high-resolution T_1_ images

In a subset of patients with post-contrast 3D T_1_ MPRAGE acquisitions, we performed an additional (semi-)quantitative analysis. From the T_1_-weighted images, anatomical standardized ROIs were identified using DL + DiReCT,^23^ a publicly available tool (https://github.com/SCAN-NRAD/DL-DiReCT) for brain anatomy segmentation, providing labels for 32 cortical parcellations per hemisphere as well as subcortical structures. Anatomical labels were generated using the ‘–model v6’ option to select a model trained with additional post-contrast images.^30^ To establish correspondence to the perfusion data, the tMIP map was rigidly registered to the T_1_-weighted image using FSL flirt^31,32^ with six degrees of freedom and mutual information as the cost function. By applying the inverse transformation matrix from the registration to the label map, the anatomical labels were moved into the space of the perfusion data. Subsequently, ROI-wise statistics for each subject were calculated from the perfusion data. Additionally, an intra-individual *z*-score for subject-wise perfusion evaluation was calculated for each ROI as follows:


$$z_{roi}=\;\frac{\mu_{roi}\;-\;\mu_{GM}}{\sigma_{GM}}$$

where *µ* and *σ* are the individual’s mean and standard deviation of the corresponding ROI and total cortical grey matter (GM), respectively.

Asymmetry indices (AI) of regions between the left hemisphere (lh) and right hemisphere (rh) were calculated as follows:


$$AI(lh,\;rh)=\;\frac{lh\;-\;rh}{lh\;+\;rh}$$

The result ranges from +1 to −1 and is 0 for completely symmetric regions. Positive values indicate higher perfusion on the left. The quantitative evaluations focused on rCBF, as in the majority of studies of perfusion changes in patients with epilepsy, quantification and statistical analysis were based on CBF.^21^

### Statistical analysis

Statistical analyses were performed using *R* with the *stats* package version 3.6.2. A significance level *α* = 0.05 was set.^33^ For categorical variables, chi-square test of independence was performed and a two-tailed Wilcoxon rank test for non-normally distributed continuous variables.

### Visual ratings of patients with quantitative analysis

Two neuroradiologists with 20 and 8 years of experience in reading perfusion MRI in epilepsy (R.W. and P.R.) independently analysed perfusion MR images and classified them as normal or abnormal (hypoperfusion or hyperperfusion) without knowing the patient’s categorization. Perfusion abnormalities were classified as TPMA if they exceeded either the vascular territories of the cerebral arteries and their respective branches or—in cases with neoplastic brain lesions—the tumour boundaries and peri-tumoural oedema. Further, we analysed if perfusion abnormalities resolved during follow-up imaging or were present at earlier exams (Supplementary Fig. 6). Inter-rater variability on the individual rating was calculated with Cohen’s *κ*.

A second rating was performed to assess the maps for regional perfusion abnormalities (frontal, parietal, temporal and occipital and insula and thalamus), if patterns of hypoperfusion or hyperperfusion were observed. Regional hyperperfusion was defined as an increase in rCBF or rCBV or a decrease in MTT in the respective lobe as visually determined on the colour-coded perfusion maps. Hypoperfusion was defined as a decreased rCBF or rCBV or an increased MTT on the colour-coded perfusion maps in the respective lobe. If perfusion was symmetric, the maps were rated as normal on visual inspections. Inter-rater variability was calculated with Cohen’s *κ*. A final rating was determined by a consensus reading of the two raters after a review of the data.

The results of the consensus rating were compared with the AI calculated with a quantitative analysis method for each lobar subregion (ROI). To compare visual ratings with quantitative AI, left-sided perfusion asymmetry (positive AI) was defined as either left hyperperfusion or right hypoperfusion, while right-sided asymmetry (negative AI) was either right hemispheric hyperperfusion or left hemispheric hypoperfusion. Every value of the asymmetry index was compared to the respective visual rating.

## Results

### Retrospective analysis of reported radiological findings

**Figure 1:**
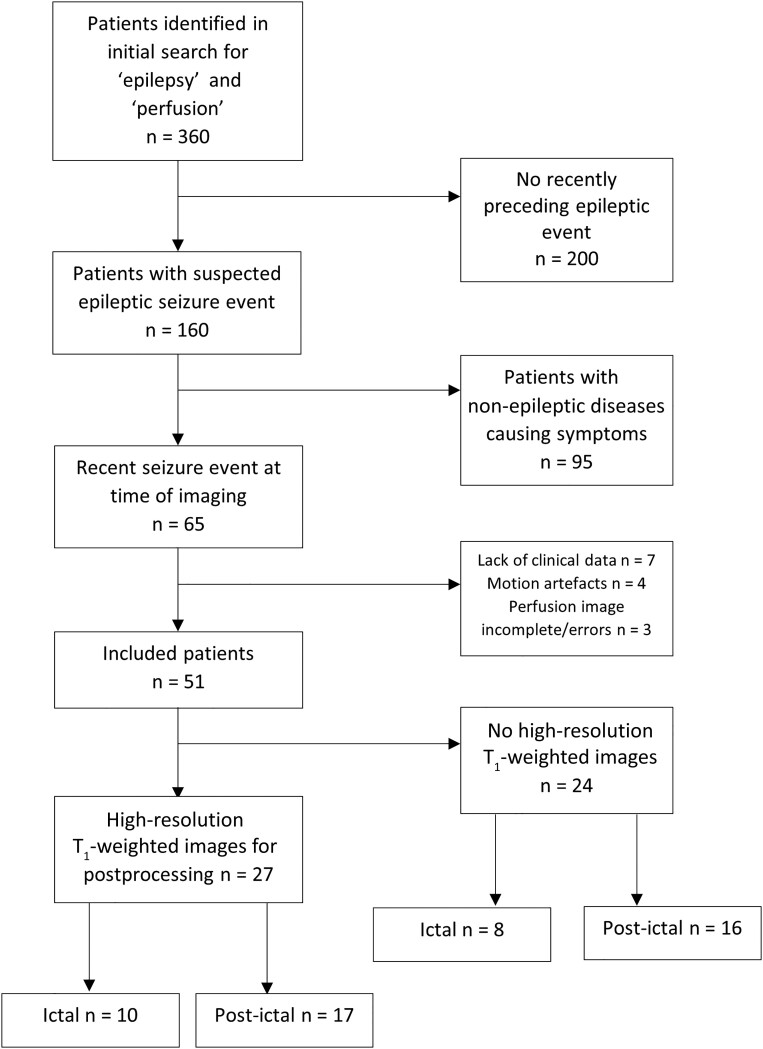
Study cohort flow chart.

The search identified 360 patients admitted between February 2008 and February 2018 (Fig. 1). Of these, 200 patients were excluded as the keyword was referring to a past event in the patient’s history, resulting in 160 patients with a recently preceding suspected seizure treated in our hospital. In 95 patients, the referral diagnosis did not match the diagnosis at discharge from the hospital (underlying conditions were seizure mimics, i.e. ischaemic stroke, cerebral haemorrhage or tumours). Out of a total of *n* = 65 patients with proven seizures at admission, seven patients were excluded due to a lack of clinical information necessary for categorization. Four patients were excluded due to motion artefacts that limited the interpretation of perfusion imaging, and three patients were excluded due to incomplete perfusion data.

Fifty-one patients were eligible for visual expert analysis (Table 1). Detailed clinical information of the individual patients is provided in Supplementary Tables 1 and 2.

**Table 1:** Retrospective analysis of reported radiological findings

Characteristic	All patients	Ictal patients	Postictal patients	*P*-value^a^
	*n* = 51	*n* = 18	*n* = 33	
**Sex**				0.2
– Female	23	11	12	
– Male	28	7	21	
Age (years)^b^	61 (46, 73) [4–88]	62 (50, 72) [44–79]	55 (42, 74) [4–88]	0.3
Time admission to MRI (h:min)^c^	02:08	01:06	02:49	0.094
	00:19–554:21	00:31–554:21	00:19–118:28	
Time from estimated onset of seizure to MRI (h:min)^c^	03:44	03:49	03:19	0.9
	01:19–139:30	01:19–139:30	01:37–30:25	
**Diffusion restriction**				0.3
– No restriction	26	7	19	
– DWI restriction	25	11	14	
**Perfusion**				<0.001
– Normal	24	0	24	
– Hypoperfusion	10	1	9	
– Hyperperfusion	17	17	0	
**Seizure type**				<0.001
– Single seizure	19	0	19	
– Seizure clusters	10	2	8	
– Status epilepticus	22	16	6	
**Seizure onset**				0.3
– Focal	44	17	27	
– Focal, no generalization	23	11	12	
– Secondary generalization	21	6	15	
– Primary generalized	6	1	15	
– Unknown	1	0	1	
**Pathologies** ^34^				0.14
– Gliosis/encephalomalacia	15	7	8	
– Developmental abnormality	1	0	1	
– Mesial temporal sclerosis	4	1	3	
– Tumours	8	5	3	
– Vascular	6	0	6	
– Unknown	8	1	7	
– Other	9	4	5	

If not otherwise indicated, data are numbers of patients. ^a^ Statistical tests between the ictal and post-ictal groups: Wilcoxon rank-sum test; chi-square test of independence, calculated *P* values to indicate significance in group differences. ^b^ Median (IQR) [age range]; *n*. ^c^ Median (h:min), time range from minimum to maximum below.

No significant differences were observed for sex, age, time of seizure onset to MRI or time from estimated seizure onset or admission to MRI between the ictal and post-ictal groups. Furthermore, pathologies also showed no statistically significant difference between the ictal and the postictal subgroup (Supplementary Table 3).

Regional cortical hyperperfusion corresponding to either the clinically symptomatogenic zone or EEG seizure focus was observed in 17/18 ictal patients (94% sensitivity, *P* < 0.001, Table 1). One patient presented with focal hypoperfusion exceeding the vascular territories, while there was no reported normal perfusion in the ictal group. Twenty-four (73%) out of 33 patients classified as postictal showed normal perfusion and nine (27%) hypoperfusion on visual analysis. A DWI restriction was observed in 11/18 of the ictal (61%) and in 14/33 (42%) of the postictal patients.

### Quantitative analysis of patients with high-resolution T_1_ images

A subgroup of 27/51 patients (53%) with post-contrast T_1_-weighted MPRAGE were eligible for quantitative analysis (Table 2). Nine of 10 (90%) ictal patients have been categorized as ictal hyperperfusion and 1 of 10 (10%) as hypoperfusion. Four out of 17 postictal patients presented with hypoperfusion (24%), and 13 (76%) patients had normal perfusion. Seven out of 10 patients had continuous epileptic activity on EEG.

**Table 2:** Demographic and clinical data of the subgroup with quantitative analysis

Characteristic	Quantitative analysis (QA) patients	QA ictal patients	QA postictal patients	*P*-value^a^
	*n* = 27	*n* = 10	*n* = 17	
**Sex**				0.008
– Female	14	9	5	
– Male	13	1	12	
Age (years)^b^	52 (35, 68)	66 (52, 72)	46 (30, 62) [4–81]	0.027
	[4–81]	[44–79]		
Time admission to MRI (h:min)^c^	02:34	02:23	02:28	0.7
	00:28–554:21	00:32–554:21	00:28–118:28	
Time from estimated onset of seizure to MRI (h:min)^c^	04:02	05:44	03:19	0.5
	01:19–139:30	01:19–139:30	01:37–30:25	
**Diffusion restriction**				> 0.9
– No restriction	12	4	8	
– DWI restriction	15	6	9	
**Perfusion**				< 0.001
– Normal	13	0	13	
– Hypoperfusion	5	1	4	
– Hyperperfusion	9	9	0	
**Seizure type**				0.003
– Single seizure	7	0	7	
– Seizure clusters	8	1	7	
– Status epilepticus	12	9	3	
**Seizure onset**				0.4
– Focal	23	10	13	
– Focal, no generalization	9	4	5	
– Secondary generalization	14	6	8	
– Primary generalized	3	0	3	
– Unknown	1	0	1	

If not indicated, data are numbers of patients. ^a^ Statistical tests between the ictal and post-ictal groups: Wilcoxon rank-sum test, chi-square test of independence and calculated *P*-values to indicate significance in in group differences. ^b^ Median (IQR) [age range]; *n*. ^c^ Median (h:min), time range from minimum to maximum below.

Median age of the ictal patients with quantitative analysis was 66 years (interquartile range 52–72) with an age range from 44 to 79 years. In the postictal group, age median was 46 years (interquartile range 30–62 years), ranging from 4 to 81 years of age, lower than in the ictal patients (*P* = 0.027). The sex mix between the ictal and postictal subgroups was also different in patients with quantitative analysis (*P* = 0.008). These differences were not observable in the entire group (*P* = 0.3 and *P* = 0.2, respectively).

Median time between estimated seizure onset and MR imaging was 5 and 44 min (ranging from 1 h 19 min to 139 h 30 min). In three individuals, seizure onset time was not known; therefore, the time ‘last seen well’ was used (Supplementary Tables 1 and 2, Patients 5, 6 and 9).

Patients in the entire cohort (Table 1, *n* = 51) were on average slightly older (61 years *P* = 0.03) compared to the QA subgroup (Table 2, *n* = 27, median age = 52 years). Otherwise, there was no statistically significant difference between the two cohorts for sex (*P* = 0.5), seizure type (*P* = 0.2), seizure onset (*P* = 0.2), time of admission to MRI (*P* = 0.2) and time from estimated onset to MRI (*P* = 0.4), as well as perfusion reports (*P* > 0.9), and reported diffusion restrictions (*P* = 0.5).

### Semi-quantitative group-wise TPMA analysis

**Figure 2:**
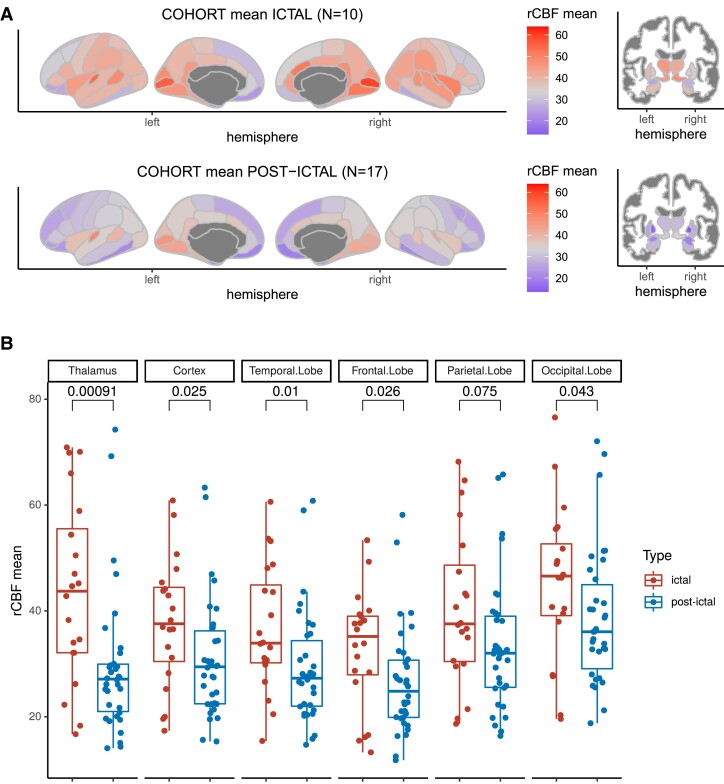
**Group-wise rCBF differences: ictal versus post-ictal** (mean and interquartile ranks). (**A**) Quantitatively evaluated maps of the averaged cohorts. Colorbars indicate the global mean of cortical rCBF across both cohorts. (**B**) Statistical differences between ictal and post-ictal cohort. Each datapoint represents the quantitative rCBF value for a region of interest (ROI) of an individual patient. p-values from a Mann-Whitney-U test.

Group-wise comparisons of mean rCBF between the ictal and postictal patient cohorts yielded increased rCBF values in the ictal cohort in the thalamus (*P* = 0.00091), temporal lobe (*P* = 0.01), whole cortex (*P* = 0.025), frontal lobe (*P* = 0.026) and occipital lobe (*P* = 0.043), as well as for a trend in the parietal lobe (*P* = 0.075) (Fig. 2B). Quantitative analysis was in general less sensitive for rCBV than for rCBF; the same statistics for rCBV are provided in Supplementary Figs 5.

The most prominent TPMA differences were identified in the thalamus (Fig. 2B). Mean rCBF increases in the thalamus were observed in the ictal cohort, in contrast to the postictal cohort where thalamic perfusion was decreased (*P* = 0.00091). Hypoperfusion in the thalamus was observed in the postictal versus hyperperfusion in the ictal cohort. The mesiofrontal and prefrontal cortex was relatively hypoperfused in both cohorts relative to the grey matter (Fig. 2A).

Subject-wise analysis of the *z*-score maps showed perfusion increase in 8 out of 10 ictal subjects (80%) in the left thalamus and 7 out of 10 (70%) in the right thalamus. In the postictal cohort, 12 out of 17 patients (71%) had negative intra-individual *z*-scores (rCBF) in the left thalamus, and 13 out of 17 patients (76%) had negative intra-individual *z*-scores (rCBF) in the right thalamus, indicating thalamic hypoperfusion.

The global mean rCBF was elevated in ictal versus postictal patients (mean rCBF 37.9 SD ± 12.0 mL g^−1^ min^−1^ versus 30.8 ± 11.2 mL g^−1^ min^−1^, respectively). The largest differences between the ictal and the postictal cohort, on the lobar level, were observed in the temporal lobe and the thalamus. Region-wise parcellations with elevated rCBF were detected predominantly in the occipital lobe, temporal lobe and frontal lobe. Parcellations with the most prominent rCBF decrease were predominantly located in the frontal lobes and temporal pole in the ictal cohort.

In postictal patients, elevated rCBF values were predominantly detected in the temporal and occipital lobe, except from the lingual gyrus instead of the cingulate gyrus. Parcellations with the most prominent rCBF decrease were detected in the temporal pole but were more pronounced in the globus pallidum and the frontal pole.

In one patient in the ictal group (Patient 10, Supplementary Tables 1 and 2) that was visually rated as hypoperfused in the left parietal lobe and normally perfused in the thalamus, quantitative analysis confirmed parietal left hypoperfusion and further revealed bilateral (left > right) thalamic hyperperfusion.

### Visual ratings and comparison to quantitative measures

At the subject level, both neuroradiologists rated TPMA with a high concordance of Cohen’s *κ* = 0.886 (Supplementary Figs 1 and 2). For the presence of hyperperfusion, the agreement was 100% between both raters while differences occurred in rating normal (agreement of 71.4%) versus hypoperfusion (agreement of 82%). Due to the complexity of the widespread perfusion abnormalities, the agreement between the two raters across all regions was low: Cohen’s *κ* = 0.365 (for ratings and confusion matrix, see Supplementary Figs 1 and 2).

**Figure 3:**
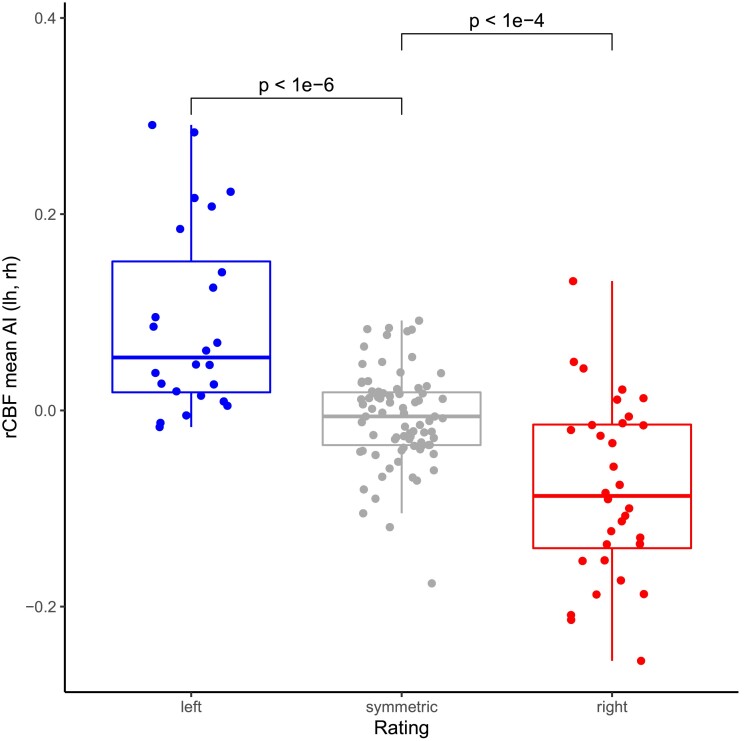
**AI and visual ratings: comparison of rCBF AI from the semi-quantitative analysis to visual ratings (consensus).** Each data point represents a ROI pooled across all subjects. The lateralization indicates if the raters considered a region to show increased perfusion on the left or right hemisphere compared to the corresponding contralateral side. ROIs with larger rCBF on the left hemisphere tend to have positive AI while ROIs with larger rCBF on the right hemisphere tend to have negative AI (as asymmetry is calculated as left minus right). *P*-values from a Mann–Whitney U test.

In a comparison of AI to visual ratings (Fig. 3), ROIs with visually higher perfusion in the left hemisphere showed positive AI (quantitative left-sided perfusion asymmetry, *P* < 10^−6^). Likewise, ROIs with visually higher perfusion in the right hemisphere showed negative AI (quantitative right-sided perfusion asymmetry, *P* < 10^−5^). ROIs with visually rated normal perfusion showed median AI close to 0 (green box). Further comparisons between visual ratings and quantitative evaluation are in Supplementary Figs 3 and 4.

### Exemplary case vignettes

**Figure 4:**
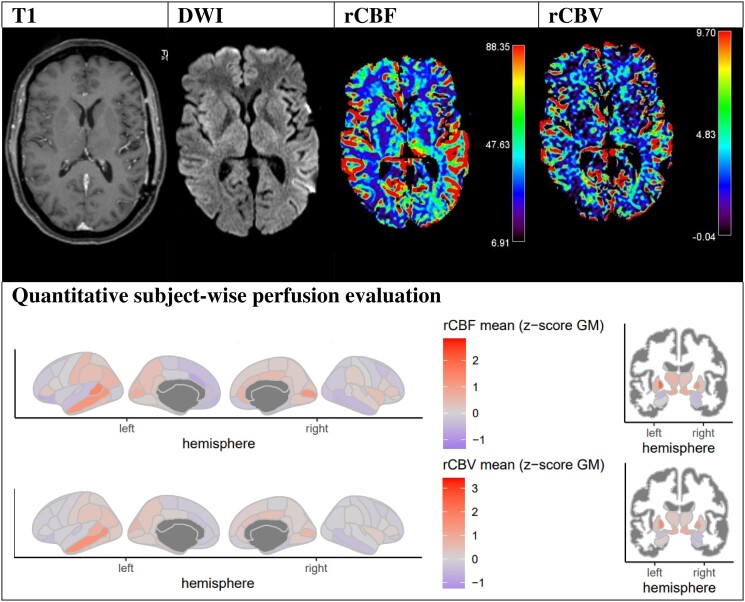
**Evaluation example of Patient 2 (Supplementary Tables 1 and 2): 48-year-old female patient with aphasia and weakness in the right arm due to cluster seizures.** DSC-MRI indicates parietal left cortical and left thalamic hyperperfusion (rCBF and rCBV).

**Figure 5:**
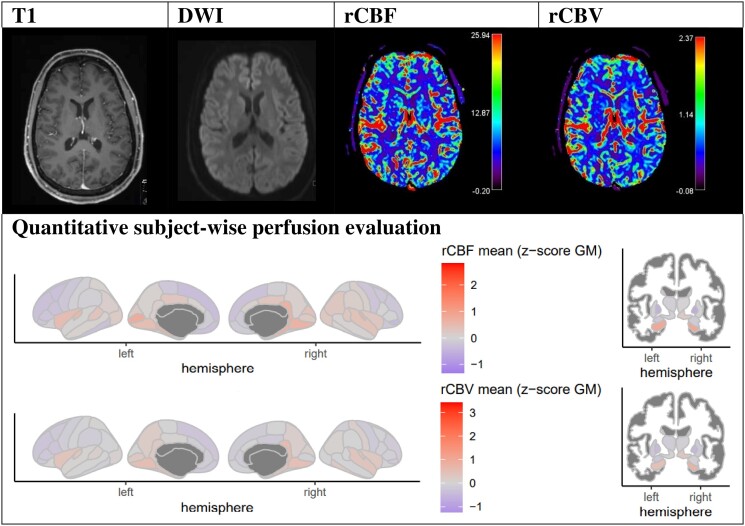
**Evaluation example of Patient 18 (Supplementary Tables 1 and 2): 30-year-old male with cluster seizures with speech arrest and repetitive extension of both arms as well as impaired consciousness.** DSC-MRI: hypoperfusion temporoparietal left (rCBF and rCBV).

To illustrate the workup of TPMA analysis with perfusion evaluation according to the clinical history of each patient, we selected two case vignettes (Figs 4 and 5) with structural T_1_-weighted image, DWI, rCBF and rCBV perfusion image slices and the corresponding quantitative subject-wise perfusion maps: Case vignette 1 and Case vignette 2.

## Discussion

In this study, we investigated TPMA in a retrospective cohort of patients who underwent advanced MRI with DWI and perfusion imaging during an emergency workup after an epileptic seizure. We investigated the diagnostic yield of TPMA to differentiate ictal and postictal patients based on visual expert ratings of regional perfusion abnormalities. We complemented the expert readings with new metrics to better visualize regional perfusion abnormalities by using a deep learning–based anatomy segmentation and extracting regional perfusion statistics that enable visualization of subtle perfusion abnormalities on a subject-wise level.^23^

Two main points stand out from this study: (i) TPMA can be reliably identified in patients who present with an NCSE or ongoing epileptic activity and undergo early MRI diagnosis in an emergency setting; and (ii) beyond variant patterns of cortical TPMA, the presence of thalamic hyperperfusion is a favourable discriminator of ictal versus postictal TPMA. While human raters performed well in the identification of global ictal versus postictal perfusion findings, accordance was low in the depiction of large-scale perfusion abnormalities beyond the core TPMAs.

Our analysis of imaging reports showed TPMA in all ictal patients, and we observed a significant association between peri-ictal imaging and corresponding hyperperfusion by previous publications.^15,35–39^ Several studies have investigated the sensitivity and time dependency of transient peri-ictal abnormalities with perfusion CT and MRI and reported regional alterations of cerebral perfusion in up to 78% patients,^21^ even in extended time frames of up to 3 days after onset of an NCSE or clusters of seizures.^40,41^ Our results corroborate previous observations that demonstrated significantly upregulated cerebral metabolism in brain areas involved in seizure generation and seizure spread. Increased CBF is a sensitive parameter to determine increased perfusion, in particular if the vascular territories are exceeded.^21,42^ In our study, visual DSC perfusion assessment revealed a sensitivity of 94% in patients with SE or seizure clusters. With 61.1% among the ictal and 42.4% among the postictal patients, we also observed a high number of focal DWI restrictions, which exceeded previous reports from a systematic review.^43^ The higher sensitivity in our cohort may be explained by our policy to perform MR imaging as early as possible and the predominance of NCSE patients in the ictal cohort.

While expert raters identified ictal perfusion patterns reliably, visual categorization of large-scale perfusion abnormalities on a lobar level or depiction of subcortical grey matter perfusion abnormalities yielded only moderate agreement. This limitation of human reading capacity is in line with a previous study that reported only moderate inter-rater concordances for patterns of spread on postictal perfusion CT.^44^ We therefore sought for a more standardized approach to compare perfusion metrics using a deep learning–based segmentation of brain tissue and intra-individual *z*-score transformation of ROI-wise extracted perfusion parameters for interindividual comparisons. Using a standardized approach, we were able to depict patterns of thalamocortical perfusion abnormalities that were able to discriminate between ictal NCSE/clusters of seizures and postictal states. This is in keeping with recent studies that suggested thalamic hyperperfusion beyond neocortical TPMA, as a new marker to diagnose NCSE using arterial spin labelling (ASL) perfusion imaging.^45–47^ One study that compared ASL with DSC-MRI, FLAIR and DWI reported thalamic perfusion abnormality in approximately half (49.0%) of patients presenting during SE.^47^ Overall, TPMA were more frequently depicted by ASL when compared to DSC-MRI (39.2%) or DWI (35.3%).^47^ The excellent diagnostic yield of ASL perfusion compared to DSC-MRI may be explained by the repetitive acquisitions in ASL versus the first pass ‘snapshot’ effect in DSC, the improved signal-to-noise ratio and insensitivity to blood vessel permeability confounds. However, when using the proposed method (qMriMaps with DL + DiReCT), we detected thalamic perfusion abnormalities in 8/10 patients with DSC-perfusion.

Thalamic hyperperfusion has been initially observed in patients with idiopathic generalized epilepsy (IGE), where a focal increase in thalamic blood flow was observed by H_2_^15^O-PET during hyperventilation-induced typical absence seizures, providing evidence for thalamocortical circuit abnormalities as the underlying pathophysiological substrate of IGE.^48^ Another early study described decreased postictal perfusion in both the temporal lobe and the ipsilateral thalamus in TLE patients with by single-photon emission computed tomography (SPECT).^49^ Subcortical peri-ictal perfusion abnormalities in basal ganglia and thalamus have been employed for seizure lateralization.^50^ Ipsilateral thalamic and basal ganglia hyperperfusion has been reported in temporal lobe epilepsy.^51^ Weder *et al*.^52^ reported subcortical peri-ictal rCBF increases after injection of the radiopharmacon in the ipsilateral thalamus 21 ± 13 s after the first clinical seizure signs. However, similar observations have only exceptionally been reported in patients during NCSE due to its unavailability under emergency conditions.^45^ Under emergency conditions, immediate availability of perfusion imaging is a prerequisite to improve decision making in patients with NCSE. In a CT study by Giovannini *et al*.,^46^ all patients with a definite NCSE showed patterns of thalamocortical hyperperfusion, emphasizing the dedicated role of perfusion imaging in emergency situations and critical care.

We also observed a trend towards thalamocortical hypoperfusion in the postictal group when semi-*quantitatively* compared with the ictal group. Due to the limited sample size, this preliminary observation of widespread postictal perfusion needs further evaluation in larger cohorts. On the large scale, we observed a regional perfusion decrease in mesial frontal areas relative to the mean rCBF in both cohorts. The findings of regional perfusion abnormalities have not been observed previously in reference analyses using H_2_^15^O-PET or ASL in healthy controls.^53,54^ The origin and relevance of this relative decrease in mesial frontal perfusion in both ictal and postictal cohorts remain to be determined. It may either reflect disconnection of functional networks induced by repetitive epileptic seizures, progressive functional decline or indicate cortical degeneration of neurovascular units (NVU) in the frontal lobe.^55^ Regional volume reductions in the mesiofrontal and prefrontal cortex in patients with chronic epilepsy have been identified in a worldwide ENIGMA study.^56^ These findings may reflect tissue loss associated with decreased metabolic demands unrelated to the seizure and may further explain similarities of the large-scale perfusion maps in ictal versus post-ictal patients.^56^

## Limitations

Despite starting our analysis with 360 patients, the rather small number of patients that could finally be included and evaluated and variant types of seizures and epilepsies as retrospectively identified from our radiology logfiles hinder conclusions related to specific epilepsy syndromes or specific pattern analyses beyond thalamocortical perfusion. High-resolution T_1_-weighted images were not available from all patients, enabling quantitative analysis only in a subgroup (*n* = 27) of our cohort. Notably, the demographics of the subgroup are comparable to the whole cohort and did not differ in most of the clinical variables. Perfusion analysis is limited to a small number of voxels in some parcellations, such as transverse temporal, frontal pole and temporal pole. Therefore, we refrained from statistical testing on the sublobar level. In our study, we did not perform a distinct discrimination analysis between focal and generalized syndromes and associated patterns of thalamic hyperperfusion or hypoperfusion due to the limited sample size. Since the ictal group consisted predominantly of NCSE patients, the findings are not applicable to patients with other epilepsy types.

## Outlook

In summary, the proposed quantitative approach has been demonstrated to detect differences in thalamocortical TPMA between patients investigated during SE and clusters of seizures on the one hand and postictal patients on the other hand with high sensitivity. The proposed deep learning approach is faster, more objective and reproducible than visual interpretation and enables standardized analysis of larger patient cohorts with respect to advanced analysis of brain network organization and association with regional neuronal degeneration.^57,58^ The subject-wise *z*-score transforms enable the fast and unbiased analysis of thalamocortical brain perfusion abnormalities on an individual level. To further strengthen the diagnostic yield of thalamocortical perfusion abnormalities, especially to discriminate conditions with persisting ictal activity that requires intervention from postictal conditions and seizure mimics, further prospective studies including also patients’ various seizure mimics such as stroke, TIA or migraine are mandatory. The proposed quantitative approach allows to investigate perfusion data systematically in a standardized way and therefore enables advanced analysis like brain network organization, which is of particular interest in epilepsy.^57^ We plan to apply the proposed method on data from a large prospective study of first-seizure patients.^58^

## Supplementary Material

fcad047_Supplementary_Data

## Data Availability

Anonymized patient data are presented in Supplementary Tables 1 and 2. Visual ratings are available in Supplementary Fig. 1. Derived data that support the findings of this study are available from the authors upon reasonable request. The pipeline to generate the proposed quantitative maps from T_1_ and corresponding perfusion MRI is publicly available at: https://github.com/SCAN-NRAD/qMriMaps.

## Abbreviations

AIF =: arterial input function
ASL =: arterial spin labelling
CBF =: cerebral blood flow
CBV =: cerebral blood volume
DSC =: dynamic susceptibility contrast
DWI =: diffusion-weighted imaging
ED =: epileptiform discharges; FLAIR = fluid-attenuated inversion recovery
IBE =: International Bureau for Epilepsy
ILAE =: International League Against Epilepsy
MTT =: mean transit time
NCSE =: non-convulsive status epilepticus
NVU =: neurovascular unit; ROI = region of interest
SE =: status epilepticus
SSE =: subtle status epilepticus
SVD =: singular value decomposition
SWI =: susceptibility-weighted imaging
TE =: echo time
Tmax =: time to maximum
tMIP =: time maximum intensity projection
ToF =: time-of-flight angiography
TPMA =: transient peri-ictal MRI abnormalities
TR =: repetition time
TTP =: time to peak
