# Supplementary material for: Large-scale transient peri-ictal perfusion magnetic resonance imaging abnormalities detected by quantitative image analysis
Source: Brain Commun. 2023 Feb 24;5(2):fcad047. doi: 10.1093/braincomms/fcad047 (PMC10012410; doi:10.1093/braincomms/fcad047)
Supplement: fcad047_Supplementary_Data [file fcad047_supplementary_data.pdf]

Supplementary material

Supplementary Table 1: Patient data for each subject. EEG features: PLEDs = periodic lateralized epileptiform discharges, ETP = epilepsy typical signals, LPDs = lateralized periodic discharges, RDA = rhythmic delta activity. The administered antiepileptic treatment is the treatment that patients received during the hospitalization because of the acute seizure event.

**Post processed Group with high resolution T1 image for coregistration**									
**Subject number**	**Age**	**Sex**	**Seizure onset**	**Seizure type**	**EEG features**	**Disease**	**Time since epilepsy diagnosis in years**	**Clinical presentation**	**Administered antiepileptic treatment**
1 EpiPerf0002_f_071	71	f	focal	nonconvulsive status epilepticus	slowed down focus left frontotemporal	oligodendroglioma	12	global aphasia and new confusion state	clonazepam, lamotrigin
2 EpiPerf0006_f_048	48	f	focal	seizure clusters	ETPs left	glioblastoma multiforme	0	aphasia and weakness of right arm	Lorazepam, valproate, lacosamide
3 EpiPerf0009_f_073	73	f	focal (secondary generalized)	nonconvulsive status epilepticus	LPDs right	herpes encephalitis	0	confusion and loss of vigilance, therapy refractory status epilepticus	lorazepam, levetiracetam, phenytoin, valproate, disoprivane
4 EpiPerf0011_f_049	49	f	focal (secondary generalized)	nonconvulsive status epilepticus	LPDs right	structural epilepsy since childhood meningitis	46	Therapy refractory Status epilepticus, Loss of consciousness, Penthotal and Disoprivan-Coma	clonazepam, levetiracetam, Pentothal, Disoprivan
5 EpiPerf0012_f_079	79	f	focal (secondary generalized)	nonconvulsive status epilepticus	slowed down focus right with delta activity	hippocampal atrophia	16	Confusion and left-sided paresis	lorazepam, levetiracetam, valproate
6 EpiPerf0014_f_062	62	f	focal (secondary generalized)	nonconvulsive status epilepticus	LPDs right and RDA	multifocal glioma	0	staring into space, head turning to the left, followed by tonic-clonic spasms of all extremities.	diazepam, propofol
7 EpiPerf0017_f_060	60	f	focal (secondary generalized)	convulsive status epilepticus	PLEDs left	structural epilepsy after multiple cvi	3	generalized tonic clonic seizure with persistent utterances motor of the right arm and aphasia	clonazepam, levetiracetam, lamotrigine
8 EpiPerf0026_f_069	69	f	focal	nonconvulsive status epilepticus	continuuous theta-delta-activity left fronto-temporal	low grade glioma	0	global aphasia, headache	clonazepam
9 EpiPerf0028_m_078	78	m	focal (secondary generalized)	nonconvulsive status epilepticus	ETPs right	traumatic brain injury	27	Loss of consciousness, left hemiparesis	levetiracetam, phenytoin, valproate, disoprivane
10 EpiPerf0034_f_044	44	f	focal	nonconvulsive status epilepticus	LPDs in EEG	multiple meningeoma	2	generalized tonic-clonic convulsions followed by aphasia and paresis of right arm	clonazepam, clobazam, levetiracetame, lamotrigine

11 EpiPerf0003_m_068	68	m	focal	single seizure	no EEG	anaplastic oligoastrocytoma	0	aphasia, turned head to the right, twitching of the right side of the face, loss of consciousness and tonic-clonic twitching of both arms	levetiracetam
12 EpiPerf0005_m_034	34	m	Primary generalized	seizure clusters	no ETPs	Epilepsy unknown origin	7	generalized tonic-clonic seizure total of 4x, left-sided mild paresis	levetiracetam
13 EpiPerf0008_m_032	32	m	focal	single seizure	no EEG	arachnoid cyst	6	Aphasia and confusion	lamotrigine
14 EpiPerf0010_m_026	26	m	focal (secondary generzalized)	nonconvulsive status epilepticus	postictal SE EEG	traumatic brain lesions	2	Loss of consciousness	clonazepam, disoprivane, levetiracetam, lamotrigine
15 EpiPerf0013_m_075	75	m	focal	seizure clusters	repetitive LPDs in long-term EEG before imaging	focal epilepsy of unknown origin	4	speech arrest, trembling of the lower jaw	phenytoin, gabapentin, levetiracetam, lamictal, clobazam
16 EpiPerf0016_f_004	4	f	Focal (secondary generalized)	nonconvulsive status epilepticus	EEG diffuse ETPs	encephalopathy of unknown origin	0	unclear loss of consciousness, fixed gaze, gaze deviation to the left	diazepam, phenobarbital, levetiracetam
17 EpiPerf0018_m_062	62	m	unknown	convulsive status epilepticus	slowed down focus frontotemporal left with no ETPs	known epilepsy of unknown aetiology, most likely genetic	7	From sleep initial confused, uncoordinated movements and tonic clonic seizure with defecation and urination, gcs 3	midazolam, levetiracetam
18 EpiPerf0020_m_030	30	m	focal (secondary generalized)	seizure clusters	repetitive ETPs long-term EEG	known temporal lobe epilepsy	6	Serial seizures with absences every minute, speech arrest, some smacking with extension of the arms.	diazepam, oxacarbazepin, clonazepam, perampanel, lamotrigin
19 EpiPerf0022_f_081	81	f	Primary generalized	seizure clusters	no EEG	primary generalised epilepsy	54	generalized tonic clonic seizure, fatigue, and Todd's palsy on the left side	clonazepam, phenobarbital
20 EpiPerf0023_m_052	52	m	Primary generalized	seizure clusters	slowed down focus left	sepsis from pneumonia under ART	0	generalized tonic-clinical seizure	clonazepam, levetiracetam
21 EpiPerf0024_m_046	46	m	focal (secondary generalized)	single seizure	slowed down focus and steeper signals right	anaplastic astrozytoma WHO III	0	generalized spasm, extension posture of arms, prolonged aphasia, gcs 8	levetiracetam
22 EpiPerf0025_f_024	24	f	focal	seizure clusters	slowed down focus temporoparietal right ETPs	Epilepsy unknown origin	0	Dysfunction of the left hand with sensory disturbances and cramps.	levetiracetam, clobazam, phenytoin, oxcarbazepin,
23 EpiPerf0027_m_025	25	m	focal (secondary generalized)	single seizure	no EEG	sleep deprivation	0	generalized tonic-clonic seizure and subsequently slowed down and disoriented	levetiracetam

24 EpiPerf0029_f_065	65	f	focal (secondary generalized)	single seizure	no EEG	hippocampal sclerosis with pharmacoresistant symptomatic epilepsy	5	bifrontal headache followed by generalized tonic clonic seizure	lorazepam, diazepam, lamotrigine
25 EpiPerf0030_f_046	46	f	focal (secondary generalized)	single seizure	no ETPs	meningioma frontal right	0	Loss of consciousness with generalized tonic clonic seizure, gcs 9	diazepam, levetiracetam
26 EpiPerf0031_m_055	55	m	focal (secondary generalized)	single seizure	no EEG	stroke residuals after traumatic brain injury	0	generalized tonic clonic seizure followed by fatigue and deceleration	levetiracetam
27 EpiPerf0032_m_036	36	m	focal	seizure clusters	no ETPs	MELAS	1	Initial twitching of the left arm, loss of consciousness with generalized twitching	lorazepam, pregabalin

Excluded patients for quantitative analysis

**Subject number**	**Age**	**Sex**	**Seizure onset**	**Seizure type**	**EEG features**	**Pathology**	**Clinical presentation**	**Administered antiepileptic treatment**
28	79	m	focal	convulsive status epilepticus	Rhythmic ETPs left hemispheric	old cvi	Twitching of mouth and right hand, aphasia	lorazepam, phenytoin, midazolam
29	61	f	focal	nonconvulsive status epilepticus	severed slowed down focus frontotemporal right	epilepsy of unknown origin	left arm paresis, hemineglect to left	levetiracetam, lacosamid
30	48	f	focal	seizure clusters	Multifocal ETPs in slowed down focus parieto-occipital right	MELAS	acute vision loss and right sided hemisyndrome, right positive babinski	clonazepam, gabapentin, lamotrigine
31	62	m	focal	nonconvulsive status epilepticus	PLEDs lefthemispheric	traumatic brain injury and SAB	aphasia, focal epileptic seizure	clonazepam, levetiracetam
32	73	m	focal	nonconvulsive status epilepticus	slowed down focus left frontotemporal	resected glioblastoma	aphasia, right sided hemisymptoma	clonazepam, lacosamid
33	47	m	Primary generalized	nonconvulsive status epilepticus	slowed down focus frontal left	genetic epilepsy	hemisymptomatic right, desorientateloss of consciousness, residual hemiparesis right	lamotrigin, natriumvalproate
34	55	m	focal	nonconvulsive status epilepticus	PLEDs occipitoparietal left	traumatic SAB brain lesions	aphasia and right sided motoric hemisyndrome	clonazepam, phenytoin, levetiracetam
35	68	m	focal	nonconvulsive status epilepticus	PLEDs frontotemporal left	multiple brain infarctions	aphasia and hemiparesis right	clonazepam, phenytoin, levetiracetam
36	82	m	focal	single seizure	no EEG	old cvi	aphasia and fixed gaze	levetiracetam
37	44	m	focal	single seizure	no ETPs, theta dominant signal	temporomesial sclerosis	comatose, GCS 10	lamotrigin, natriumvalproate

38	52	m	focal	single seizure	no EEG	resected astrocytoma	aphasia and right sided hemisyndrome	levetiracetam
39	74	f	focal (secondary generalized)	single seizure	slowed down focus left temporal	mutiple slcerosis	generalized tonic clonic seizure, residual paresis left arm	clonazepam, levetiracetam
40	68	m	focal (secondary generalized)	single seizure	no EEG	traumatic SAB, SDH and craniotomy	aphasia an right sided hemiparesis	diazepam
41	67	f	Primary generalized	single seizure	generalized ETPs (spike-waves)	known generalized epilepsy	acute confusion, drooping left sided mouth-corners	lorazepam, levetiracetam, natriumvalproate
42	79	m	focal (secondary generalized)	nonconvulsive status epilepticus	no EEG	microangiopathy	generalized tonic clonic status epilepticus with loss of consciousness, left sided residual hemiparesis	diazepam
43	65	f	focal (secondary generalized)	seizure clusters	low voltage EEG matching a postictal state	demential developments	loss of consciousness, hemiparesis right side	n/a
44	50	f	Primary generalized	single seizure	no EEG	multiple, hyperintense juxtacortical and periventricular white matter lesions	generalized tonic clonic movements, tonguebite left	lamotrigin
45	75	m	focal	single seizure	repetitive ETPs in long term EEG	lacunary change in centrum semiovale right	aphasia, twitching of the mouth	phenytoin, gabapentin, lamotrigin, levetiracetam
46	88	m	focal	single seizure	no ETPs, diffuse beta waves	vascular leucencephalopathy	aphasia, increased salivation	levetiracetam
47	49	f	focal (secondary generalized)	single seizure	pathologic focus frontotemporal left	intracranial bleeding temporal left	loss of consciousness and muscle contractions	levetiracetam
48	77	m	focal (secondary generalized)	single seizure	no EEG	vascular leucencephylopathy	generalized tonic clonic movements, tonguebite, loss of urine, loss of consciousness	levetiracetam
49	75	f	focal (secondary generalized)	nonconvulsive status epilepticus	slowed down focus right frontocentral with spike wave	resected meningeoma	loss of consciousness, residual	clonazepam

					complexes - NCSE		hemisyndrome left	
50	61	f	focal	single seizure	no EEG	leucencephalopathy	unresponsive with preserved alertness	levetiracetam
51	42	m	focal	nonconvulsive status epilepticus	slowed down focus temporofrontal right	severe head trauma with SDH, SAB and contusion bleeding	prolongued brachiocephal hemisyndrome left after focal status epilepticus frontotemporal right	lorazepam, levetiracetam

Supplementary Table 2: Information of the categorization as well as times for the enrolled patients 1 to 52.

Subject number	MRI Timing	Perfusion (0 = normal, 1= hypo, 2 = hyper)	Side of reported Perfusion abnormality (left, right or symmetrical)	Corresponding Diffusionsrestriction (DR) reported 1 = reported DR 0 = no corresponding DR reported	Clinical lateralization	Lateralization on EEG	Time Admission to MRI (h:m)	Time from estimated onset to MRI (h:m)	Admission to hospital Time	MRI aquisition Time	EEG Recording Time	Estimated symptom onset
**Post processed Group with high resolution T1 image for coregistration**												
1 EpiPerf0002_f_071	ictal	2	left	0	left	left	02:34	03:44	19.10h Day 1	21.44h Day 1	11.12h Day 2	18.00h Day 1
2 EpiPerf0006_f_048	ictal	2	left	0	left	left	00:32	01:19	22.17h Day 1	22.49h Day 1	02.22h Day 2	21.30h Day 1
3 EpiPerf0009_f_073	ictal	2	right	1	no	right	04:26	11:26	17.00h Day 1	21.26h Day 1	19.22h Day 1	10.00-12:00h Day 1
4 EpiPerf0011_f_049	ictal	2	right	1	no	right	554:21	139:30	12.00h Day 1	15.21h Day 23	18.30h Day 23	22.00-24.00h Day 17
5 EpiPerf0012_f_079	ictal	2	right	1	right	right	02:12	07:27	21.15h Day 1	23.27h Day 1	15.42h Day 2	16.00h Day 1
6 EpiPerf0014_f_062	ictal	2	right	0	no	right	45:25	50:36	17.36h Day 1	15.01h Day 3	15.36h Day 3	15.00-17.00h Day 1
7 EpiPerf0017_f_060	ictal	2	left	1	left	left	12:20	17:33	23.13h Day 1	11.33h Day 2	09.17h Day 2	18.00-20.00h Day 1
8 EpiPerf0026_f_069	ictal	2	left	1	left	left	00:36	01:25	09.04h Day 1	09.40h Day 1	13.06h Day 1	08:15h Day 1
9 EpiPerf0028_m_078	ictal	2	right	0	right	right	01:03	04:02	23.59h Day 1	01.02h Day 2	03.46h Day 2	21.00-23.00h Day 1
10 EpiPerf0034_f_044	ictal	1	left	1	left	left	00:36	01:40	18.39h Day 1	19.15h Day 1	15.52h Day 2	17.35h Day 1
11 EpiPerf0003_m_068	postictal	0	symmetrical	0	left	n/a	02:48	03:19	17.31h Day 1	20.19h Day 1	no EEG	17.00h Day 1
12 EpiPerf0005_m_034	postictal	0	symmetrical	0	right	no lateralization	00:45	01:51	21.06h Day 1	21.51h Day 1	23.54h Day 1	20.00h Day 1
13 EpiPerf0008_m_032	postictal	0	symmetrical	0	left	n/a	04:00	05:24	20.54h Day 1	00.54h Day 2	no EEG	19.30h Day 1
14 EpiPerf0010_m_026	postictal	0	symmetrical	0	right	no lateralization	04:05	30:25	16.20h Day 2	20.25h Day 2	21.57h Day 2	14:00h Day 1

15 EpiPerf0013_m_075	postictal	0	symmetrical	0	no lateralization	no lateralization	118:28	04:33	12.05h Day 1	10.33h Day 6	07.00-13:00h Day 6	06:00h Day 6
16 EpiPerf0016_f_004	postictal	0	symmetrical	0	no lateralization	left	00:55	03:07	11.12h Day 1	12.07h Day 1	14.46h Day 1	09.00-10.00h Day 1
17 EpiPerf0018_m_062	postictal	0	symmetrical	1	no lateralization	left	16:03	16:33	00.30h Day 1	16.33h Day 1	14.29h Day 1	00.00h Day 1
18 EpiPerf0020_m_030	postictal	1	left	1	left	left	65:50	03:12	18.22h Day 1	12.12h Day 4	09.00h Day 4	09.00h Day 4
19 EpiPerf0022_f_081	postictal	1	right	1	right	n/a	01:16	03:13	14.12h Day 1	15.28h Day 1	no EEG	12.15h Day 1
20 EpiPerf0023_m_052	postictal	0	symmetrical	0	no lateralization	left	48:01	04:42	20.41h Day 1	20.42h Day 3	16.51h Day 3	16.00h Day 3
21 EpiPerf0024_m_046	postictal	0	symmetrical	1	left	right	00:46	02:06	15.10h Day 1	15.56h Day 1	19.53h Day 1	13.50h Day 1
22 EpiPerf0025_f_024	postictal	0	symmetrical	1	right	right	03:43	05:03	09.20h Day 1	13.03h Day 1	15.21h Day 1	08.00h Day 1
23 EpiPerf0027_m_025	postictal	0	symmetrical	1	no lateralization	n/a	06:08	06:50	19.12h Day 1	01.20h Day 2	no EEG	18.30h Day 1
24 EpiPerf0029_f_065	postictal	1	right	0	no lateralization	n/a	01:40	02:49	21.09h Day 1	22.49h Day 1	no EEG	20.00h Day 1
25 EpiPerf0030_f_046	postictal	0	symmetrical	1	no lateralization	n/a	01:01	01:37	10.56h Day 1	11.57h Day 1	no EEG	10.20h Day 1
26 EpiPerf0031_m_055	postictal	0	symmetrical	1	left	n/a	02:10	02:48	19.08h Day 1	21.18h Day 1	no EEG	18.30h Day 1
27 EpiPerf0032_m_036	postictal	1	left	1	left	n/a	01:54	06:37	07.43h Day 1	09.37h Day 1	no EEG	03.00-06.00h Day
**Excluded patients for quantitative analysis**												
28	ictal	2	left	0	left	left	00:35	03:22	18.47 Day 1	19.22 Day 1	21.22 Day 1	16.00 Day 1
29	ictal	2	right	1	right	right	06:24	07:08	17.44 Day 1	00:08 Day 2	09:18 Day 2	17.00 Day 1
32	ictal	2	right	1	left	right	00:31	01:41	17.10 Day 1	17.41 Day 1	20.09 Day 1	16.00 Day 1
31	ictal	2	left	0	left	left	00:23	03:54	11:46 Day 1	12.09 Day 1	12.53 Day 1	08.15 Day 1
32	ictal	2	left	1	left	left	00:29	03:23	23.09 Day 1	23.38 Day 1	13.18 Day 2	20.15 Day 1
33	ictal	2	left	1	left	left	01:07	05:05	12.28 Day 1	13.35 Day 1	15.20 Day 2	08.30 Day 1
34	ictal	2	left	1	left	left	01:05	03:17	02.42 Day 1	03.47 Day 1	07.49 Day 1	00.30 Day 1
35	ictal	2	left	0	left	left	01:16	02:01	09.30 Day 1	10.46 Day 1	12.14 Day 1	08.45 Day 1
36	postictal	0	symmetrical	0	left	n/a	00:26	02:42	16.16 Day 1	16.42 Day 1	8.25 Day 4	14.00 Day 1
37	postictal	0	symmetrical	1	no lateralization	no lateralization	02:50	04:40	09.50 Day 1	12.40 Day 1	10.34 Day 1	08.00-09.00 Day 1
38	postictal	1	left	0	left	n/a	00:19	03:04	13.15 Day 1	13.34 Day 1	no EEG	10.30 Day 1

39	postictal	0	symmetrical	0	no lateralization	left	20:28	21:36	18.08 Day 1	14.36 Day 2	08.55 Day 2	17.00 Day 1
40	postictal	0	symmetrical	0	left	n/a	04:01	05:11	15.20 Day 1	19.21 Day 1	no EEG	14.10 Day 1
41	postictal	1	left	1	no lateralization	left	01:25	02:30	12.05 Day 1	13.30 Day 1	14.34 Day 1	11.00 Day 1
42	postictal	0	symmetrical	0	right	n/a	01:37	02:56	23.49 Day 1	01.26 Day 2	no EEG	22.30 Day 1
43	postictal	0	symmetrical	0	left	left	00:33	01:52	09.19 Day 1	09.52 Day 1	10.49 Day 1	08.00 Day 1
44	postictal	0	symmetrical	0	no lateralization	n/a	01:27	02:19	13.52 Day 1	15.19 Day 1	no EEG	13.00 Day 1
45	postictal	1	left	0	left	no lateralization	94:28	02:33	12.05 Day 1	10.33 Day 5	07.00-13.00 Day 5	08.00 Day 1
46	postictal	1	left	1	left	no lateralization	05:30	10:12	12.42 Day 1	18.12 Day 1	14.59 Day 1	08.00 Day 1
47	postictal	1	left	0	no lateralization	left	01:30	03:47	22.17 Day 1	23.47 Day 1	11.21 Day 2	20.00 Day 1
48	postictal	1	right	1	no lateralization	n/a	05:52	06:50	08.58 Day 1	14.50 Day 1	no EEG	08.00 Day 1
49	postictal	0	symmetrical	0	right	right	02:08	03:48	15.40 Day 1	17.48 Day 1	20.12 Day 1	14.00 Day 1
50	postictal	0	symmetrical	1	no lateralization	n/a	05:24	06:11	18.17 Day 1	23.41 Day 1	no EEG	17.30 Day 1
51	postictal	1	right	0	right	right	95:30	02:05	18.35 Day 1	18.05 Day 5	11.22 Day 5	16.00 Day 1

Commentary Table 1 and 2:

In three patients (4, 6, 7, Supplementary tables 1 and 2) from the ictal cohort MRI was performed later than 12 hours after estimated seizure onset. Patient 4 was under long-term penthotal and clonazepam treatment intravenous and neuroimaging performed was after clonazepam dose reduction. The EEG three hours after imaging showed continuous focal nonconvulsive status epilepcticus. Patient 6 was also in propofol and diazepam-induced coma due to a nonconvulsive status epilepticus. In MRI perfusion there was the suspicion of ongoing NCSE which was confirmed in EEG 30 minutes later. Patient 7 was admitted in a convulsive status epilepticus where motor expressions ended after clonazepam and levetiracetam treatment. The patient's vigilance worsened over night and a sensomotoric aphasia persisted. The next morning EEG showed persisting status epilepticus (periodic lateralized discharges) what lead to the respective MRI.
In these 3 cases with long time delay between onset and MRI, MRI was a selective examination after an emergency imaging workup with initial CT scanning and due to persisting epileptic activity.
Six of 51 patients (14.8%) were in-house referrals to MRI who presented with a seizure during hospitalization. In these cases time from estimated seizure onset to MRI was shorter than time from admission to MRI.

Supplementary Table 3: Summary of the diseases and pathologies according to Hakami et. al. [1]. Basic informations in Table 2 (disease).

**Abnormalities as possible seizure aetiology**				
		Total	ictal	postictal
**Gliosis/encephalomalacia**		**15**	**7**	**8**
	Poststroke	6	3	3
	Posttraumatic	6	3	3
	Postoperative tumor resection	3	1	2
**Tumors**		**8**	**5**	**3**
	Meningeoma	2	1	1
	Low grade glioma	2	1	
	High grade glioma	4	3	2
**Developmental abnormality**		**1**		**1**
	Arachnoid cyst	1		1
**Vascular**		**6**		**6**
	Leucencephalopathy/microangiopathy	5		5
	Intracranial hemorrhage	1		1
**Mesial temporal sclerosis**		**4**	**1**	**3**
**Other**		**9**	**4**	**5**
	dementical developments	1		1
	Mitochondriopathy	2	1	1
	Inflammation	3	2	1
	Primary generalized epilepsy	3	1	2
**Unknown**		**8**	**1**	**7**
		**51**	**18**	**33**

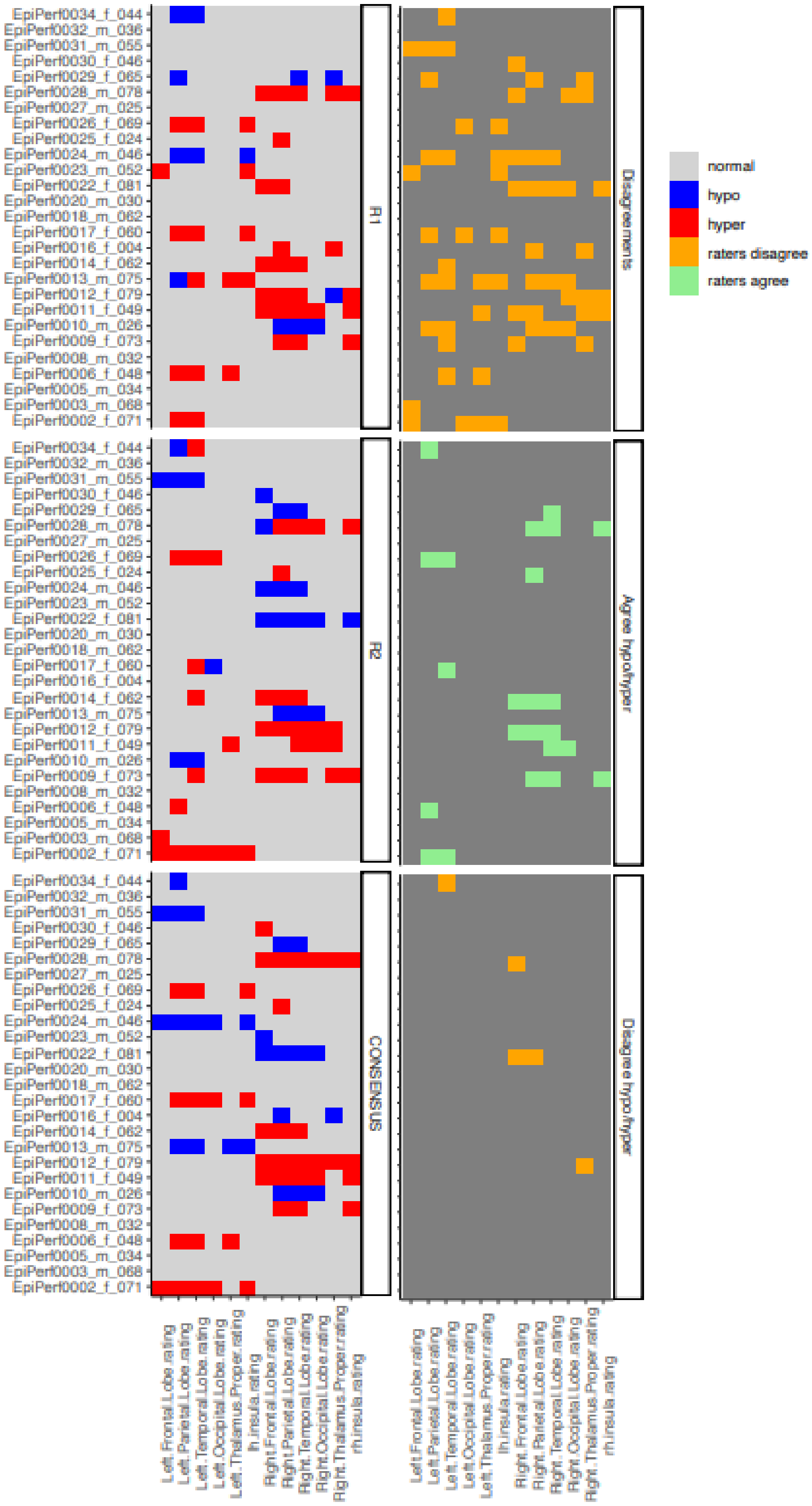

Supplementary Figure 1: Rating overview for the two raters and their consensus (left column) with hypo-perfused ROI in blue and hyper-perfused in red. Right column shows disagreements across all ROI (1st row) and agreements for ROIs with a perfusion abnormality (2nd row). Contradicting agreements were observed for a total of 5 ROI (3rd row).

A) Inter-rater agreement patient-level

Kappa = 0.886

	normal	hypo	hyper
normal	5	1	0
hypo	1	9	0
hyper	0	0	11

B) Inter-rater agreement ROI-level

Kappa = 0.365

	normal	hypo	hyper
normal	239	17	14
hypo	9	2	2
hyper	17	3	21

Supplementary Figure 2: Confusion matrix and inter-rater agreement (Cohen's kappa) on a patient-level (A) and ROI-level (B). Kappa was calculated in R with the package *caret* version 6.0-03.

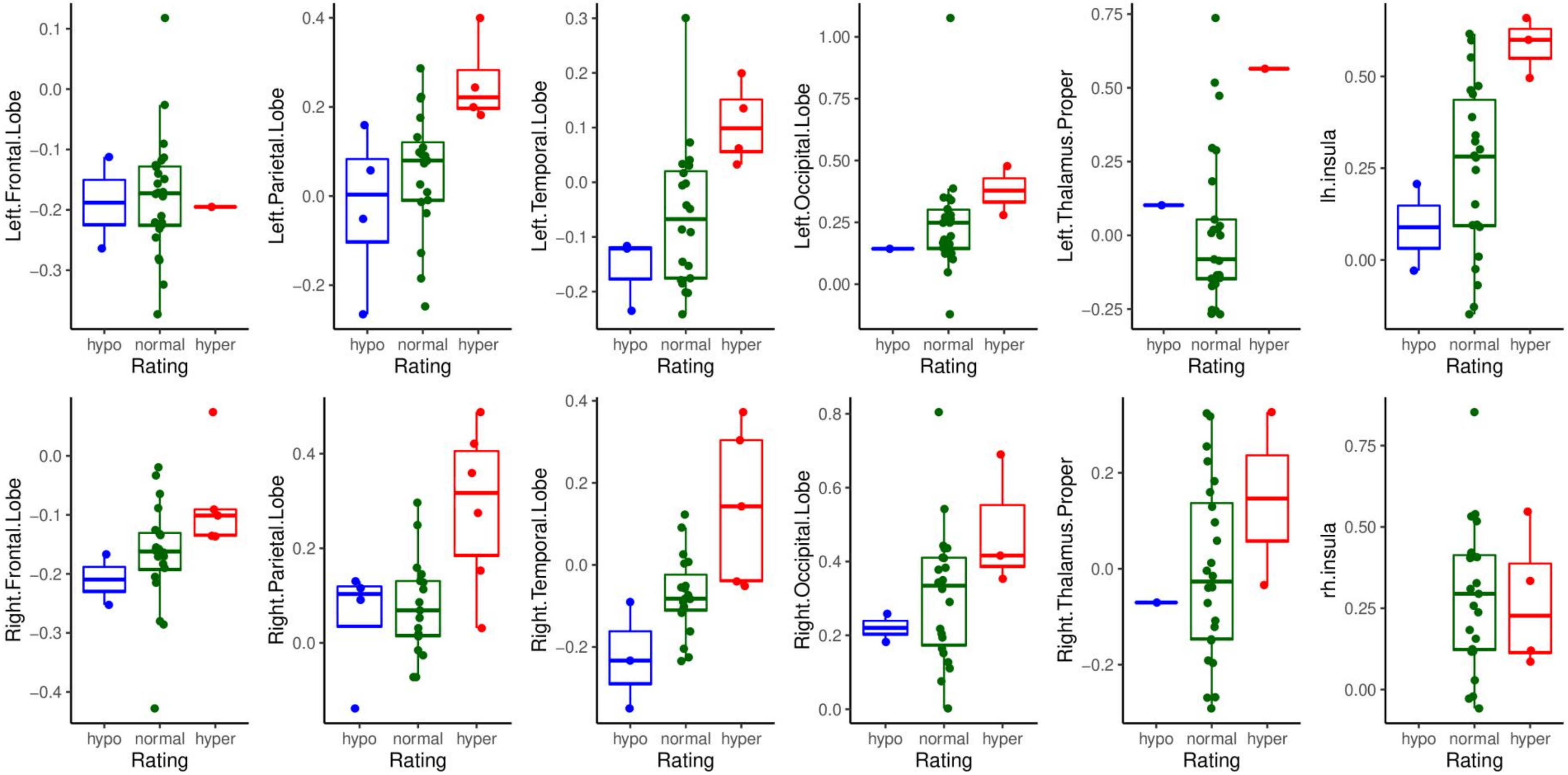

Supplementary Figure 3: Comparison of visual ratings (x-axis) with intra-individual z-scores of rCBF values (y-axis). Visual ratings for hypoperfusion are visualized in blue data points and boxplots, while visual hyperperfusion ratings are visualized in red color. The rCBF values are displayed on the x-axis. Whiskers of the boxplots extend either to the smallest/largest datapoint or span 1.5 x interquartile range, whatever is smaller. No statistical test was performed due to low sample size in each category.

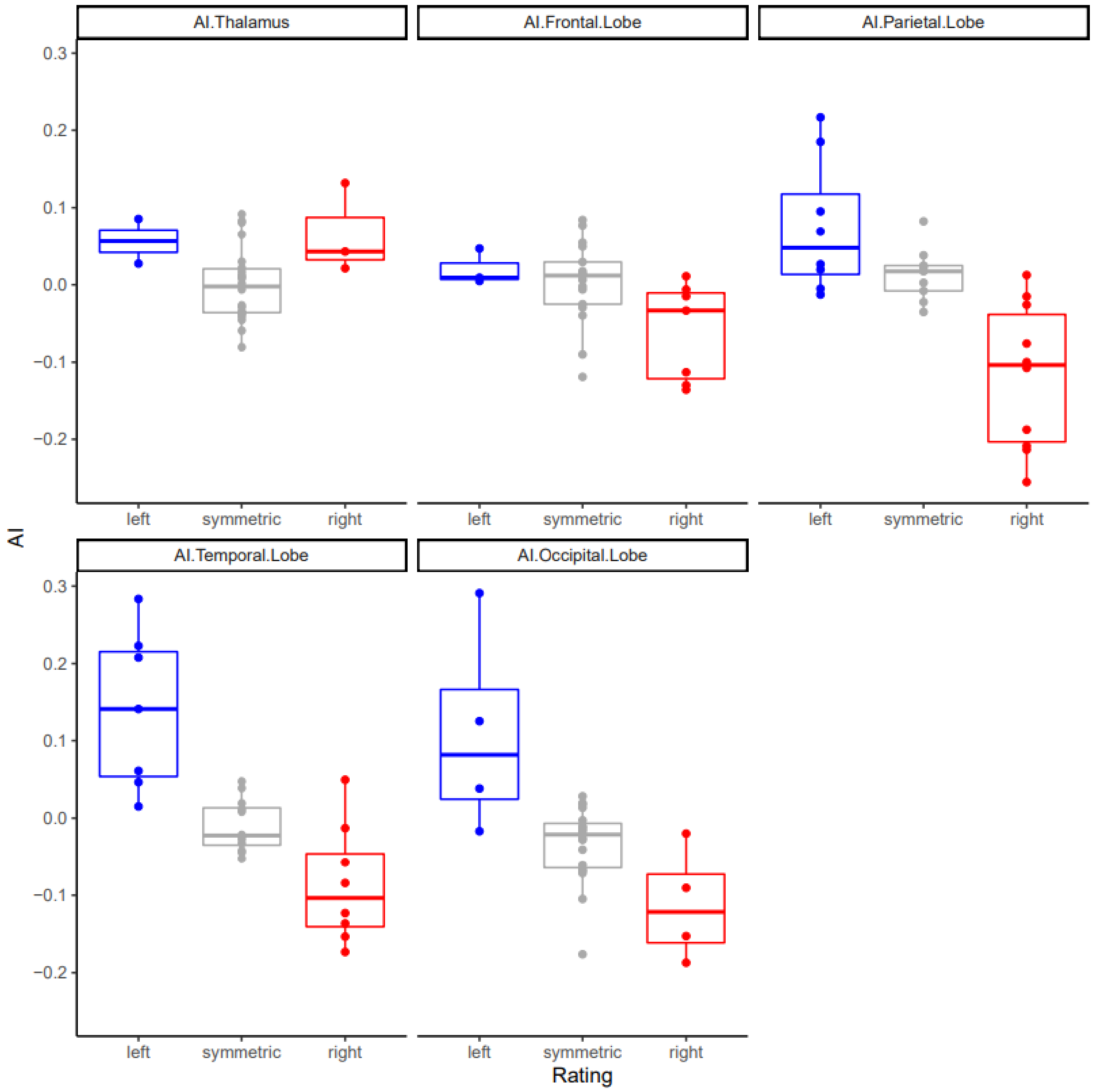

Supplementary Figure 4: Comparison of visual ratings (x-axis) with Asymmetry Indices (AI, y-axis) of rCBF values for each lobe. Visual ratings were considered: left = ROI on left hemisphere hyper- or right hemisphere hypo-perfused, symmetric = left/right equal, right = right hemisphere hyper- or left hemisphere hypo-perfused). Whiskers of the boxplots extend either to the smallest/largest datapoint or span 1.5 x interquartile range, whatever is smaller. No statistical test was performed due to low sample size in each category.

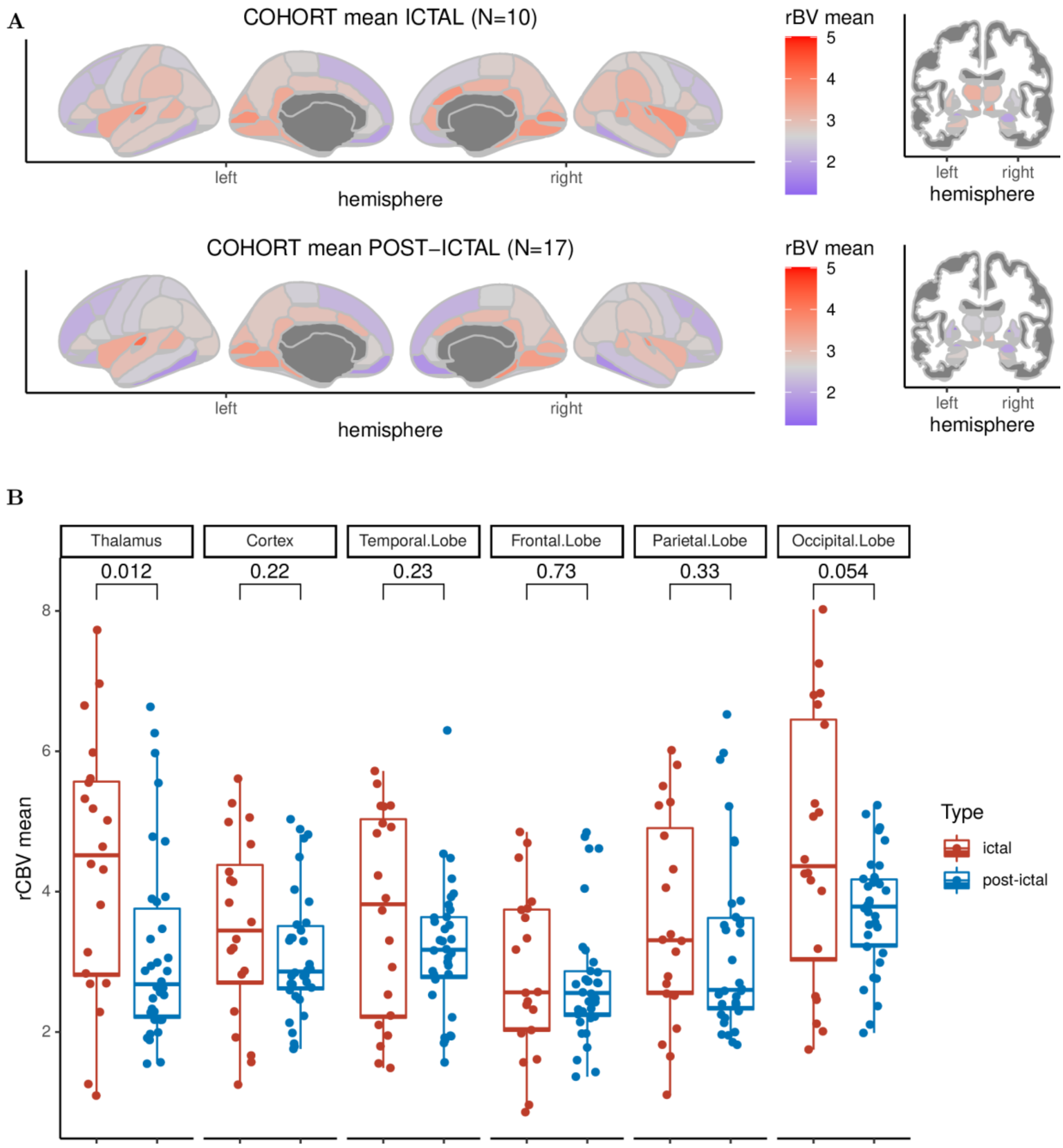

Supplementary Figure 5: Cohort statistics for the ictal and post-ictal groups using rCBV values on a ROI-level (top) and lobe-level (bottom). Each datapoint represents the quantitative rCBV value for each ROI of an individual patient. The corresponding figures using rCBF is shown in the main text in Figure 2. p-values from a Mann-Whitney-U test.

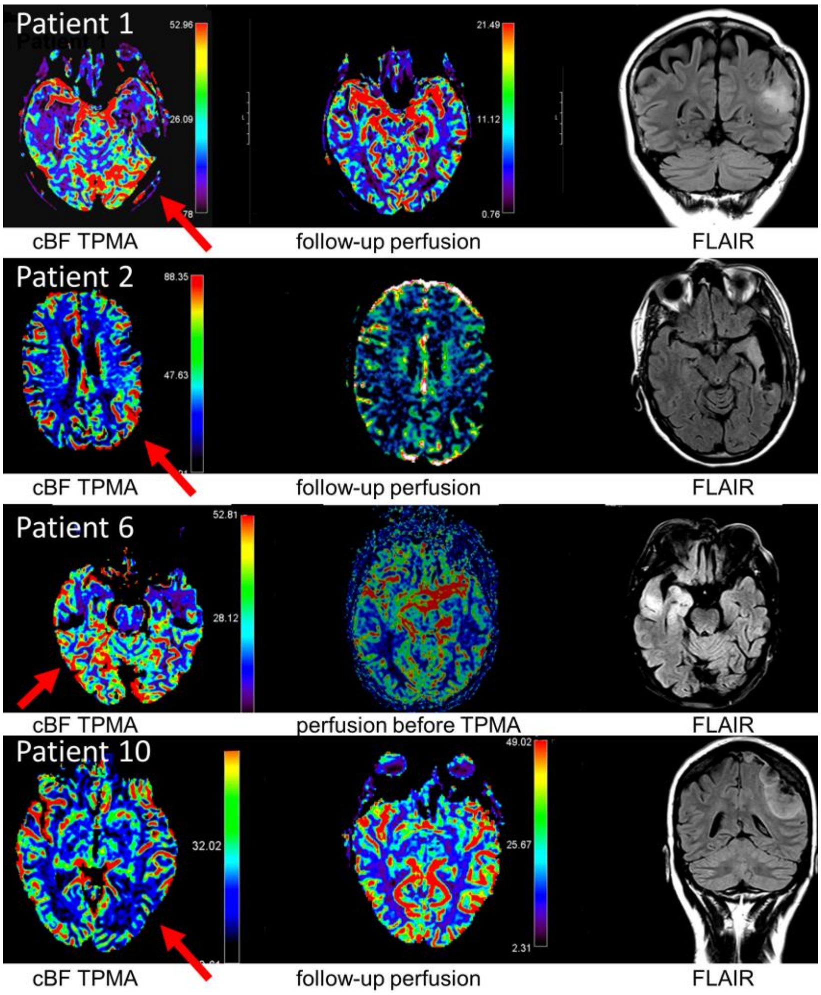

Supplementary Figure 6:
The figure illustrates TPMA in patients with tumor lesions with previous or follow-up perfusion imaging. The perfusion changes are not associated to the lesions itself.

Supplementary Reference:

1. Hakami T, McIntosh A, Todaro M, et al. MRI-identified pathology in adults with new-onset seizures. *Neurology*. Sep 3 2013;81(10):920-7. doi:10.1212/WNL.0b013e3182a35193
